# Medical Ethnobiology and Ethnopharmacology in Latin America 2013

**DOI:** 10.1155/2014/576382

**Published:** 2014-01-22

**Authors:** Ulysses Paulino Albuquerque, Edwin L. Cooper, Maria Franco Trindade Medeiros, Rômulo Romeu da Nóbrega Alves, Ana H. Ladio

**Affiliations:** ^1^Biology Department, Federal Rural University of Pernambuco, Rua Dom Manoel de Medeiros, Recife, PE, Brazil; ^2^Laboratory of Comparative Neuroimmunology, Department of Neurobiology, David Geffen School of Medicine at UCLA, Los Angeles, CA, USA; ^3^Campus Cuité, Centro de Educação e Saúde (CES), Unidade de Educação (UAE), Curso de Ciências Biológicas, Universidade Federal de Campina Grande (UFCG), Olho D'Água da Bica S/N, Cuité, PB, Brazil; ^4^Departamento de Biologia, Universidade Estadual da Paraíba, Monteiro, PB, Brazil; ^5^Laboratorio Ecotono, Universidad de Comahue, Bariloche, Argentina

Ethnobiology is a rapidly growing science, especially in Latin America. Undoubtedly, growth in this field has many explanations, including the diversity in the lines of research, the continuous training of researchers, and the great biological and cultural diversity in Latin America. This special issue contains 14 manuscripts covering different approaches in ethnobiology. We should highlight the fact that most of the papers are focused on the pharmacological evaluation of plants or animals used in traditional medical practices.

Of these 14 manuscripts, eight address the evaluation of the biological activities of medicinal plants, while two evaluate the biological activity of a number of animals used for medicinal purposes in Northeastern Brazil. Additionally, at least three papers in this special issue explore the relationship between healthcare and both cultural practices and foods. In addition, one paper employs a relatively simple approach to bring attention to the wide variety of medicinal plants that are used in Brazil, the diversity of which is possibly overestimated by taxonomic misconceptions.

Thus, we hope that this collection of papers, accompanying the annual issue of “*Medical Ethnobiology and Ethnopharmacology in Latin America*,” will give our readers valuable insights into diverse areas of ethnobiology research.

*Ulysses Paulino Albuquerque* *Ulysses Paulino Albuquerque* *Edwin L. Cooper* *Edwin L. Cooper* *Maria Franco Trindade Medeiros* *Maria Franco Trindade Medeiros* *Rômulo Romeu da Nóbrega Alves* *Rômulo Romeu da Nóbrega Alves* *Ana H. Ladio* *Ana H. Ladio*

